# Identification of animal behavioral strategies by inverse reinforcement learning

**DOI:** 10.1371/journal.pcbi.1006122

**Published:** 2018-05-02

**Authors:** Shoichiro Yamaguchi, Honda Naoki, Muneki Ikeda, Yuki Tsukada, Shunji Nakano, Ikue Mori, Shin Ishii

**Affiliations:** 1 Integrated Systems Biology Laboratory, Graduate School of Informatics, Kyoto University, Sakyo, Kyoto, Japan; 2 Laboratory of Theoretical Biology, Graduate School of Biostudies, Kyoto University, Yoshidakonoecho, Sakyo, Kyoto, Japan; 3 Data-driven Modeling Team, Research Center for Dynamic Living Systems, Graduate School of Biostudies, Kyoto University, Yoshidakonoecho, Sakyo, Kyoto, Japan; 4 Group of Molecular Neurobiology, Graduate School of Science, Nagoya University, Furoucho, Chikusa, Nagoya, Aichi, Japan; UNITED KINGDOM

## Abstract

Animals are able to reach a desired state in an environment by controlling various behavioral patterns. Identification of the behavioral strategy used for this control is important for understanding animals’ decision-making and is fundamental to dissect information processing done by the nervous system. However, methods for quantifying such behavioral strategies have not been fully established. In this study, we developed an inverse reinforcement-learning (IRL) framework to identify an animal’s behavioral strategy from behavioral time-series data. We applied this framework to *C*. *elegans* thermotactic behavior; after cultivation at a constant temperature with or without food, fed worms prefer, while starved worms avoid the cultivation temperature on a thermal gradient. Our IRL approach revealed that the fed worms used both the absolute temperature and its temporal derivative and that their behavior involved two strategies: directed migration (DM) and isothermal migration (IM). With DM, worms efficiently reached specific temperatures, which explains their thermotactic behavior when fed. With IM, worms moved along a constant temperature, which reflects isothermal tracking, well-observed in previous studies. In contrast to fed animals, starved worms escaped the cultivation temperature using only the absolute, but not the temporal derivative of temperature. We also investigated the neural basis underlying these strategies, by applying our method to thermosensory neuron-deficient worms. Thus, our IRL-based approach is useful in identifying animal strategies from behavioral time-series data and could be applied to a wide range of behavioral studies, including decision-making, in other organisms.

## Author summary

Understanding animal decision-making has been a fundamental problem in neuroscience and behavioral ecology. Many studies have analyzed the actions representing decision-making in behavioral tasks, in which rewards are artificially designed with specific objectives. However, it is impossible to extend this artificially designed experiment to a natural environment, as in the latter, the rewards for freely-behaving animals cannot be clearly defined. To this end, we sought to reverse the current paradigm so that rewards could be identified from behavioral data. Here, we propose a new reverse-engineering approach (inverse reinforcement learning), which can estimate a behavioral strategy from time-series data of freely-behaving animals. By applying this technique on *C*. *elegans* thermotaxis, we successfully identified the respective reward-based behavioral strategy.

## Introduction

Animals develop behavioral strategies, a set of sequential decisions necessary for organizing appropriate actions in response to environmental stimuli, to ensure their survival and reproduction. Such strategies lead animals to their preferred states and provide them with effective solutions to overcome difficulties in a given environment. For example, foraging animals are known to optimize their strategy to most efficiently exploit food sources [1]. Therefore, understanding behavioral strategies of biological organisms is important from biological, ethological, and engineering point of views.

A number of studies have recorded the behavioral sequences reflecting the overall animal strategies. However, mechanistic descriptions are different from phenomenological descriptions of recorded behaviors [2], and there is no well-established method that can objectively identify behavioral strategies, a mechanistic component of behavior. From a theoretical viewpoint, this mechanistic component corresponds to an algorithmic/representational level of understanding of information processing systems [3]. To derive behavioral strategies from quantitative time-series behavioral data, we propose a new computational framework based on the concept of reinforcement learning (RL).

**Fig 1:**
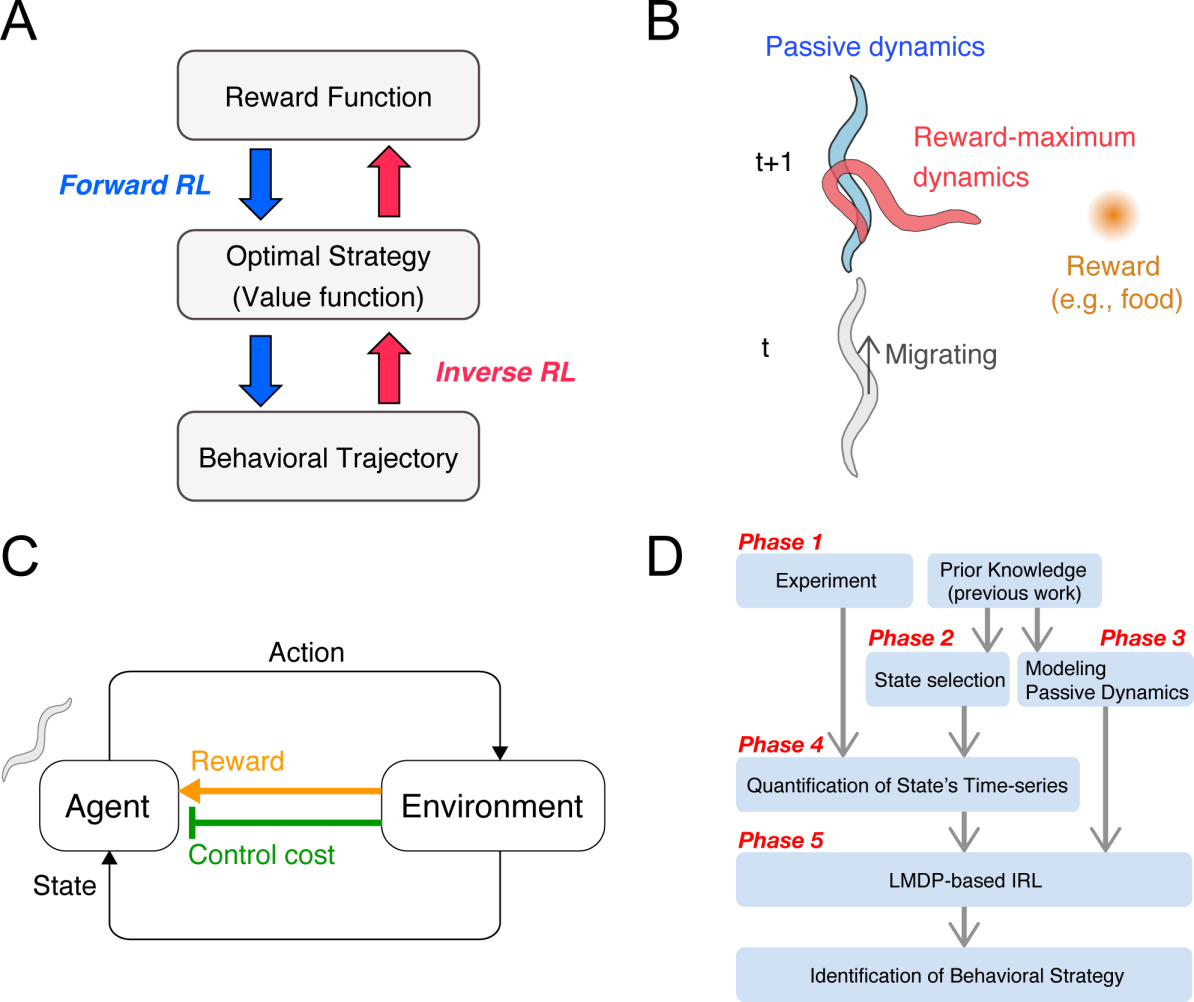
Concept and procedure of the inverse reinforcement learning (IRL)-based approach. **(A)** Reinforcement learning represents a forward problem, in which a behavioral strategy is optimized to maximize the cumulative reward given as a series of states and rewards. IRL represents an inverse problem, in which a behavioral strategy, or its underlying value and reward functions, is estimated in order to reproduce an observed series of behaviors. The behavioral strategy is evaluated by the profiles of the identified functions. **(B)** Examples of passive and controlled dynamics. An animal migrates upwards, while the food (reward) is placed to its right. In this situation, if the animal continues to migrate upwards, the distance to the food increases. If the animal exercises harder body control, that is, changes its migrating direction towards the food, the distance to the food decreases. The animal should therefore make decisions based on balancing these two dynamics. **(C)** The agent-environment interaction. The agent autonomously acts in the environment, observes the resultant state-transition through its sensory system, and receives not only a state reward but also a body control cost. The behavioral strategy is optimized to maximize the accumulation of the net reward, which is given as the state reward minus the body control cost. **(D)** IRL framework for the identification of animal behavioral strategies. If a certain behavioral strategy is under investigation, a behavioral experiment is initially performed (**phase 1**), which can either involve a free-movement task or a conditional task. Subsequently, the states and passive dynamics, based on which the animal develops its strategy, are selected and modelled (**phase 2 and 3**). For these phases, prior knowledge on the type of sensory information an animal processes is useful for appropriately selecting the states and passive dynamics. **Phases 4 and 5** involve the quantification of the time-series of the selected states and the implementation of the linearly-solvable Markov decision process-based IRL, respectively, in order to estimate the value function. The behavioral strategy can be then identified.

RL is a mathematical paradigm that represents how animals adapt their behavior to maximize cumulative rewards via trial and error [4] (blue arrow in **Fig 1A**). A previous study indicated that dopamine activity reflects the reward prediction error [5], similar to temporal difference learning in RL [6], suggesting that RL-based regulation underlies animal’s behavioral learning. Even in the simple neural circuits of *Caenorhabditis elegans* (*C*. *elegans*), dopamine-dependent activity, involved in explorative behavior, is reminiscent of RL [7]. Thus some behavioral strategies are likely associated with the reward system.

Inverse reinforcement learning (IRL) is a recently developed machine-learning framework that can solve the inverse problem of RL (blue arrow in **Fig 1A**) and estimate reward-based strategies from behavioral time-series data [8,9]. One engineering application of IRL is apprenticeship learning. For example, seminal studies on IRL employed a radio-controlled helicopter, for which the state-dependent rewards of an expert were estimated by using time-series observations of both a human expert’s manipulation and the helicopter’s state. Consequently, autonomous control of the helicopter was achieved by (forward) RL, by utilizing the estimated rewards [10,11]. This engineering application prompted studies on animal behavioral strategies by using IRL. Recently, IRL application studies have emerged, mostly regarding human behavior, with a particular interest in constructing artificially intelligent systems that mimic such behavior [12–15]. In these studies, the behavioral tasks were designed with specific objectives, thus the observed behavioral strategies were usually expected. However, IRL applications involving freely behaving animals, in a more natural environment, are far from established.

In an effort to apply IRL to freely behaving animals, we chose thermotaxis in *C*. *elegans* as a model for a behavior that is regulated by specific strategies. When worms are cultivated at a constant temperature with plenty of food and then placed on a thermal gradient without food, they show an appetitive response to the cultivation temperature [16,17]. In contrast, if they are first cultivated at a constant temperature without food and then transferred on the thermal gradient, they show an aversive behavior towards the cultivation temperature [18,19]. Although the worms are not aware of the spatial temperature profile or their current location, it is obvious that they somehow make rational decisions, depending on their feeding status. Although there are multiple potential strategies that can theoretically lead animals to their goals, the actual ones they utilize in each condition are largely unknown due to the stochastic nature of behavioral sequences, which conceals the principles of behavioral regulation, as in the case of many other animal behaviors.

In this study, we developed a new IRL framework to identify the behavioral strategy as a value function. The value function represented the benefit of each state, namely, how much future rewards were expected starting from a given state. By applying this IRL framework to time-series behavioral data of freely migrating *C*. *elegans*, we identified the value functions underlying thermotactic strategies. Fed animals behaved based on sensory information of both the absolute and temporal derivative of temperature, and their behavior involved two modes; directed migration (DM) towards the cultivation temperature and isothermal migration (IM) along contour at constant temperature. Starved worms, in contrast, used only the absolute temperature but not its temporal derivative for escaping the cultivation temperature. By further applying the IRL to thermosensory neuron-impaired worms, we found that the so-called “AFD” neurons are fundamental for the DM exhibited by the fed worms. Thus, our framework can reveal the most preferable/optimal state for the animals and, more importantly, how animals reach that state, thereby providing clues for understanding the computational principles in the nervous system.

## Results

### IRL framework

To identify animal behavioral strategies based on IRL, we initially made the assumption that they are the result of the balance between two factors: passive dynamics (blue worm in **Fig 1B**) and reward-maximizing dynamics (red worm in **Fig 1B**), which correspond to inertia-based and purpose-driven body movements, respectively. For example, even if a worm moving in a straight line wants to make a purpose-driven turn towards a reward, it cannot turn suddenly, due to the inertia of its already moving body. Thus, it is reasonable to consider that the animal’s behavior is optimized by taking the above two factors into account, i.e., by minimizing the resistance to passive dynamics and maximizing approach to the destination (reward). Such a behavioral strategy has recently been modeled by using a linearly-solvable Markov decision process (LMDP) [20], in which the agent requires not only a state-dependent reward, but also a control cost for quantifying resistance to passive dynamics (**Fig 1C**). Importantly, the optimal strategy in the LMDP is analytically obtained as the probability of controlled state transition [20]:

$$\pi \left(s_{t+1}|s_{t}\right)\propto P(s_{t+1}|s_{t})\exp\left\{v(s_{t+1})\right\},$$ (1)

where *s*_*t*_ indicates the animal’s state at time step *t*; *v*(*s*) is the value function and is defined as the expected sum of state-dependent rewards, *r*(*s*), and negative control cost, *KL*[*π*(⋅|*s*)||*p*(⋅|*s*)], from state *s* towards the future; and *P*(*s*_*t+*1_|*s*_*t*_) represents the probability of uncontrolled state transition, indicating the passive dynamics from *s*_*t*_ to *s*_*t+*1_. In this equation, the entire set of *v*(*s*) represents the behavioral strategy. Thus, the identification of a behavioral strategy is equivalent to the estimation of the value function *v*(*s*), based on the observed behavioral data (*s*_*1*_, *s*_*2*_,…*s*_*t*_,…*s*_*T*_; red arrow in **Fig 1A**). For this purpose, we used the maximum likelihood estimation (MLE) method [21]. Notably, in this estimation, we introduced a constraint to make the value function smooth, since animals generate similar actions in similar states. This constraint was essential to stably estimate the behavioral strategy of animals. The different phases of the IRL framework are depicted in the flowchart of **Fig 1D**. Following this flowchart, we applied the IRL framework to freely-migrating *C*. *elegans* under a thermal gradient.

### Phase 1: Monitoring animal behaviors

**Fig 2:**
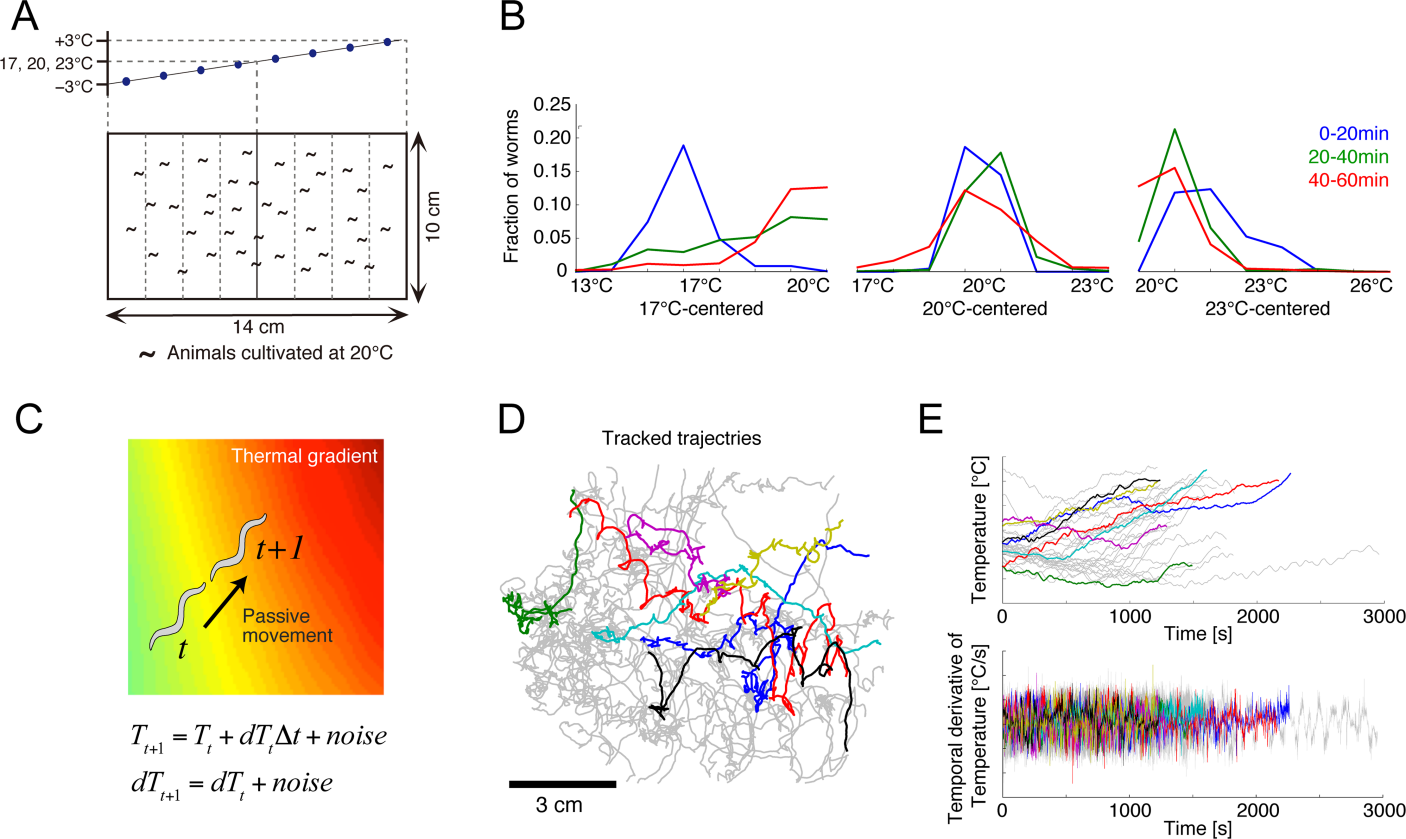
Thermotactic behavior in *C*. *elegans*. **(A)** Thermotaxis assays including a thermal gradient. In each assay, a linear temperature gradient was set along the agar surface, whose center was set at either 17, 20, or 23°C. At the onset of the assay, fed or starved worms were placed at the center of the agar surface. **(B)** Temporal changes in the spatial distribution of the fed worms under the 17°C-, 20°C- and 23°C-centered thermal gradients. **(C)** Passive dynamics of persistent migration on a linear thermal gradient. **(D)** Representative trajectories of worms extracted by the multi-worm tracking system (n = 33 in this panel). Different colors indicate individual worms. **(E)** Time series of the temperature and its derivative experienced by the migrating worms shown in C (colors correspond to those in D).

To identify the behavioral strategy underlying the thermotactic behavior of *C*. *elegans*, we performed population thermotaxis assays, in which 80–150 worms, which had been cultivated at 20°C, were placed on the surface of an agar plate with controlled thermal gradients (**Fig 2A**). Since the rate of physical contact is low at this worm density, behavioral crosstalk was negligible. To collect behavioral data, we prepared three different thermal gradients of 14–20, 17–23, and 20–26°C, centered at 17, 20, and 23°C, respectively; we expected that the first gradient would encourage ascent up the gradient, the second movement around the center, and the third descent down the gradient. Indeed, the fed worms aggregated around the standard cultivation temperature (20°C) in all gradients (**Fig 2B**).

### Phase 2: Selection of states

We first defined the worms’ state, signified by *s* in Eq (1), taking into account that it should represent the sensory information that the worms process during thermotaxis. Previous studies have shown that thermosensory AFD neurons encode the temporal derivative of temperature [22,23]; therefore, we assumed that worms select appropriate actions based not only on temperature, but also on its temporal derivative. We thus represented state by a two-dimensional (2-D) sensory space: *s* = (*T*, *dT*), where *T* and *dT* denote temperature and its temporal derivative, respectively. This means that the value function in Eq (1) represents a function of *T* and *dT*, i.e., *v*(*s*) = *v*(*T*, *dT*). Notably, we did not select the spatial coordinates on the assay plate for state, since the worms cannot recognize the spatial temperature profile or their current position on the plate.

### Phase 3: Modeling passive dynamics

Next, we defined passive dynamics, signified by *P*(*s*_*t+*1_|*s*_*t*_) in Eq (1). Passive dynamics are the result of state transitions as a consequence of uncontrolled behavior. We assumed that a worm likely migrates in a persistent direction, but in a sometimes fluctuating manner. During state transition in a short time interval, the local thermal gradient can be considered as linear (**Fig 2C**). Thus, we modelled the passive transition from state *s*_*t*_ = (*T*_*t*_, *dT*_*t*_), at time *t*, to the next state, *s*_*t+*1_ = (*T*_*t+*1_, *dT*_*t+*1_), at time *t* + 1, where *dT*_*t+*1_ maintains *dT*_*t*_ with noise perturbation, while *T*_*t+*1_ is updated as *T*_*t*_*+dT*_*t*_ with noise perturbation. Accordingly, *P*(*s*_*t+*1_|*s*_*t*_) was simply expressed by a normal distribution (please note the distinction between *T* and *t* throughout this paper).

### Phase 4: Quantification of state time-series

To quantify thermosensory states selected in phase 2, we tracked the trajectories of individual worms over 60 min within each gradient, by using a multi-worm tracking software [24] (**Fig 2D**). We then recorded the temperature that each individual worm experienced at each time-point (upper panel in **Fig 2E**) and calculated the temporal derivative of temperature by using a Savitzky-Golay filter [25] (lower panel in **Fig 2E**). State trajectories in the *T*-*dT* space were also plotted (**S2A Fig**).

### Phase 5: Identification of behavioral strategy by IRL

Using the collected state time-series data, *s* = (*T*, *dT*), and passive dynamics, *P*(*s*_*t+*1_|*s*_*t*_), we performed IRL, i.e., we estimated the value function, *v*(*s*). We modified an existing estimation method called OptV [21], by introducing a smoothness constraint, and confirmed that this constraint was indeed effective in accurately estimating the value function, when applied to artificial data simulated by Eq (1) (**S1 Fig**). Since this method could powerfully estimate a behavioral strategy based on artificial data, we next applied it to the behavioral data of the fed worms.

**Fig 3:**
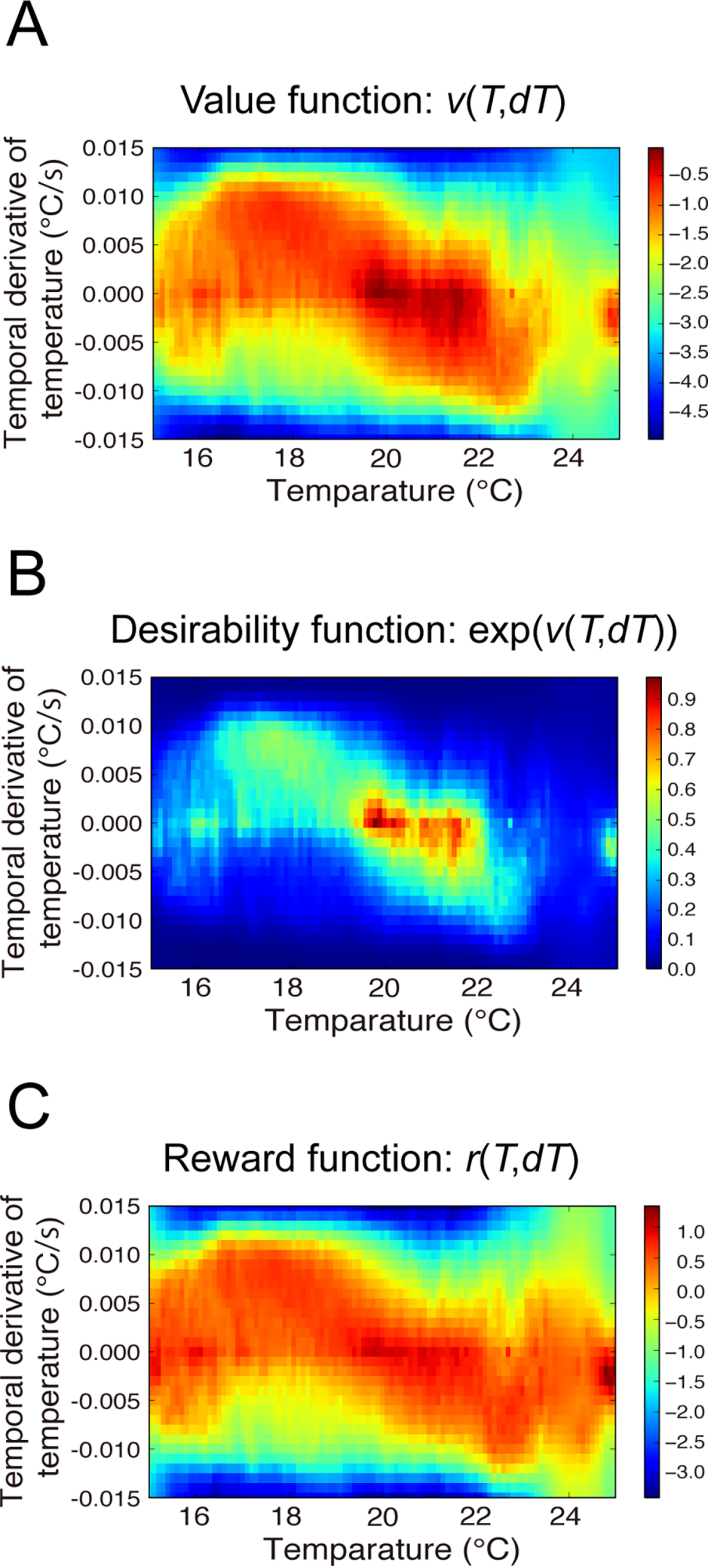
Behavioral strategy identified for fed WT worms. The behavioral strategies of the fed WT worms, as represented by the value **(A)**, desirability **(B)**, and reward **(C)** functions. The worms prefer and avoid the red- and blue-colored states, respectively.

Our method successfully estimated the value function (**Fig 3A**) and visualized the desirability function, expressed by exp(*v*(*T*, *dT*)) [21] (**Fig 3B**). Furthermore, we could calculate the reward function from the identified desirability function using Eq (8) (**Fig 3C**). The reward function primarily represents the worms’ preference, while the desirability function represents the behavioral strategy and is thus a result of optimizing the cumulative sum of rewards and negative control costs. Therefore, our method quantitatively clarified the behavioral strategy of fed *C*. *elegans*.

### Interpretation of the identified strategy

Since both the value and desirability functions essentially represented the same thermotactic strategy, we focus on the results only for the desirability function. We found that the identified desirability function peaked at *T* = 20°C and *dT* = 0°C/s, encouraging the worms to reach and stay close to the cultivation temperature. Moreover, we recognized both diagonal and horizontal components (**Fig 3B**), though the latter one was partially truncated by data limitation and data inhomogeneity (**S2B Fig**). The diagonal component represented directed migration (DM), a strategy that enables worms to efficiently reach the cultivation temperature. At lower temperatures than the cultivation temperature a more positive *dT* is favored, whereas at higher temperatures a more negative *dT* is favored. This DM strategy is consistent with the observation that worms migrate toward the cultivation temperature, and also clarifies how they control their thermosensory state throughout migration. On the other hand, the horizontal component represented isothermal migration (IM), which explains a well-known characteristic of worms, called isothermal tracking; worms typically exhibit circular migration under a concentric thermal gradient [17]. Although we used a linear, not a concentric gradient in our thermotaxis assay, our IRL algorithm successfully extracted the isothermal tracking-related migration strategy, which worked both at the cultivation temperature and at other temperatures. The desirability function (**Fig 3B**) described the strategy of state transition (Eq (1)), while the state distribution of *T* and *dT* (**S2B Fig**) was an outcome of the strategy; therefore, the desirability function was not equivalent to the actual state distribution.

During thermotaxis, worms alternate between ‘runs’ and ‘sharp turns’, which correspond to persistent migration with slight changes in direction, during long intervals, and intermittent directional changes with large angle, during short intervals, respectively [26]. Because the number of data points obtained during the runs is much larger than those during the sharp turns in total, our IRL framework could recapitulate the strategy for shallow but not for sharp turns. Indeed, we could not find a relationship between the desirability function and the rate of sharp turns (**S2C and S2D Fig**).

### Reliability of the identified strategy

**Fig 4:**
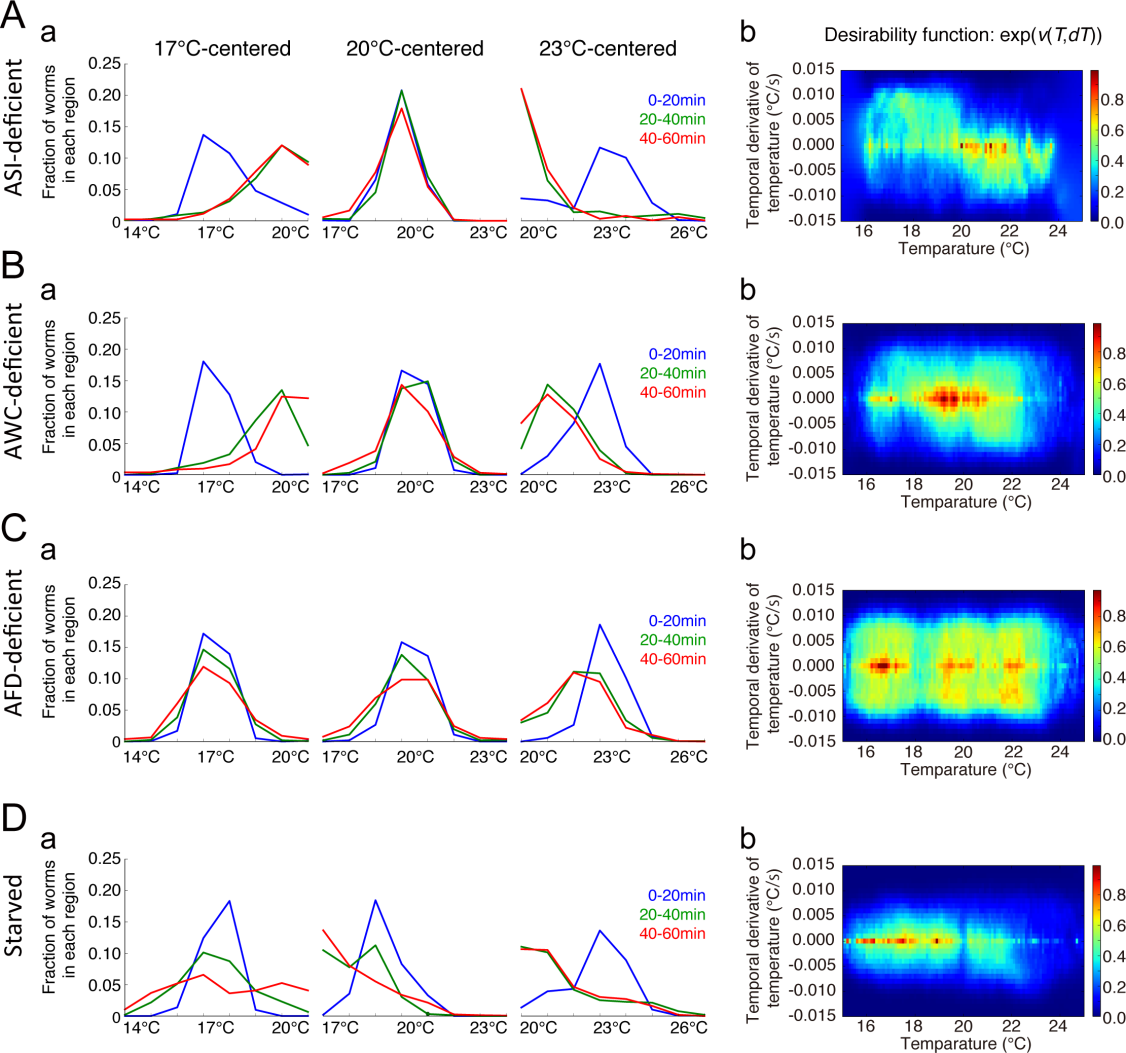
Inverse reinforcement learning analyses of ASI-, AWC-, and AFD-neuron deficient worms and starved worms. Temporal changes in distributions of ASI-, AWC-, and AFD-neuron deficient worms, as well as of starved worms in the 17°C-, 20°C- and 23°C-centered thermal gradients after behavior onset are presented in column **a** of A, B, C, and D, respectively. The corresponding desirability functions are shown in column **b** of A, B, C, and D, respectively. Starved worms disperse under a thermal gradient, while ASI- and AWC-deficient worms migrate to the cultivation temperature, similarly to fed WT worms; AFD-deficient worms show cryophilic thermotaxis.

We verified the reliability of the identified strategies with the following four ways. First, we examined the dimension of the strategy. We performed IRL based on a one-dimensional (1-D) state representation, i.e., *s* = (*T*). Comparing 1-D and 2-D cases, we used cross-validation to confirm that the prediction ability for a future state transition was significantly higher in the 2-D than in the 1-D behavioral strategy (*p =* 0.0002; Mann-Whitney U test) (**S3 Fig**). This result indicates that fed worms utilized sensory information of both the absolute temperature and its temporal derivative for their behavioral strategy. Second, we confirmed that our IRL approach recapitulated the nature of thermotactic behaviors. We simulated temperature trajectories starting from 15, 20, and 25°C, by sampling the state transition from Eq (1), using the identified value function. The simulated worm population converged around the cultivation temperature (**S4 Fig**), showing that the identified strategy indeed represented the thermotactic property of the fed worms. Third, we statistically tested the identified DM and IM strategies. As a null hypothesis, we assumed that the worms randomly migrated under a thermal gradient with no behavioral strategy. By means of surrogate method-based statistical testing, we showed that the DM and IM strategies could not be obtained by chance, indicating that both strategies reflected an actual strategy of thermotaxis (**S5 Fig**). Finally, we cross-checked the DM and IM strategies by repeating our IRL protocol on another *C*. *elegans* strain. To this end, we used worms in which the chemosensory ASI neurons were genetically ablated via cell-specific expression of caspases [27]. This ASI-deficient strain appeared to show normal thermotaxis (**Fig 4Aa**), suggesting that the ASI neurons were not responsible for thermotaxis in our assay. We found clear diagonal and horizontal components in the desirability function, supporting the existence of the DM and IM strategies (**Fig 4Ab**).

### Strategies of thermosensory neuron-deficient worms

To examine the role of the thermosensory circuit in the observed behavioral strategy, we created two worm strains in which one of the two types of thermosensory neurons, AWC or AFD, [16,17,28] had been genetically ablated via cell-specific expression of caspases. The AWC-deficient worms appeared to show normal thermotaxis (**Fig 4Ba**). The desirability function, obtained as for wild type (WT) animals (**Fig 4Bb**), suggested that AWC neurons did not play an essential role in thermotaxis. In contrast, AFD-deficient worms demonstrated cryophilic thermotaxis (**Fig 4Ca**). The desirability function consistently increased as temperature decreased (**Fig 4Cb**) but lacked the *dT-*dependent structure, indicating that the DM strategy observed in WT worms had disappeared. Moreover, the fact that AFD neurons encode the temporal derivative of temperature [22,23] further corroborates the loss of the *dT-*dependent structure. Thus, AFD-deficient worms inefficiently aimed for lower temperatures by a strategy primarily depending on the absolute temperature but not on its temporal derivative (**Fig 4Cb**). Taken together, these findings demonstrate that AFD and not AWC neurons are essential for efficiently navigating towards the desired/cultivation temperature.

### Strategy of starved worms

**Fig 5:**
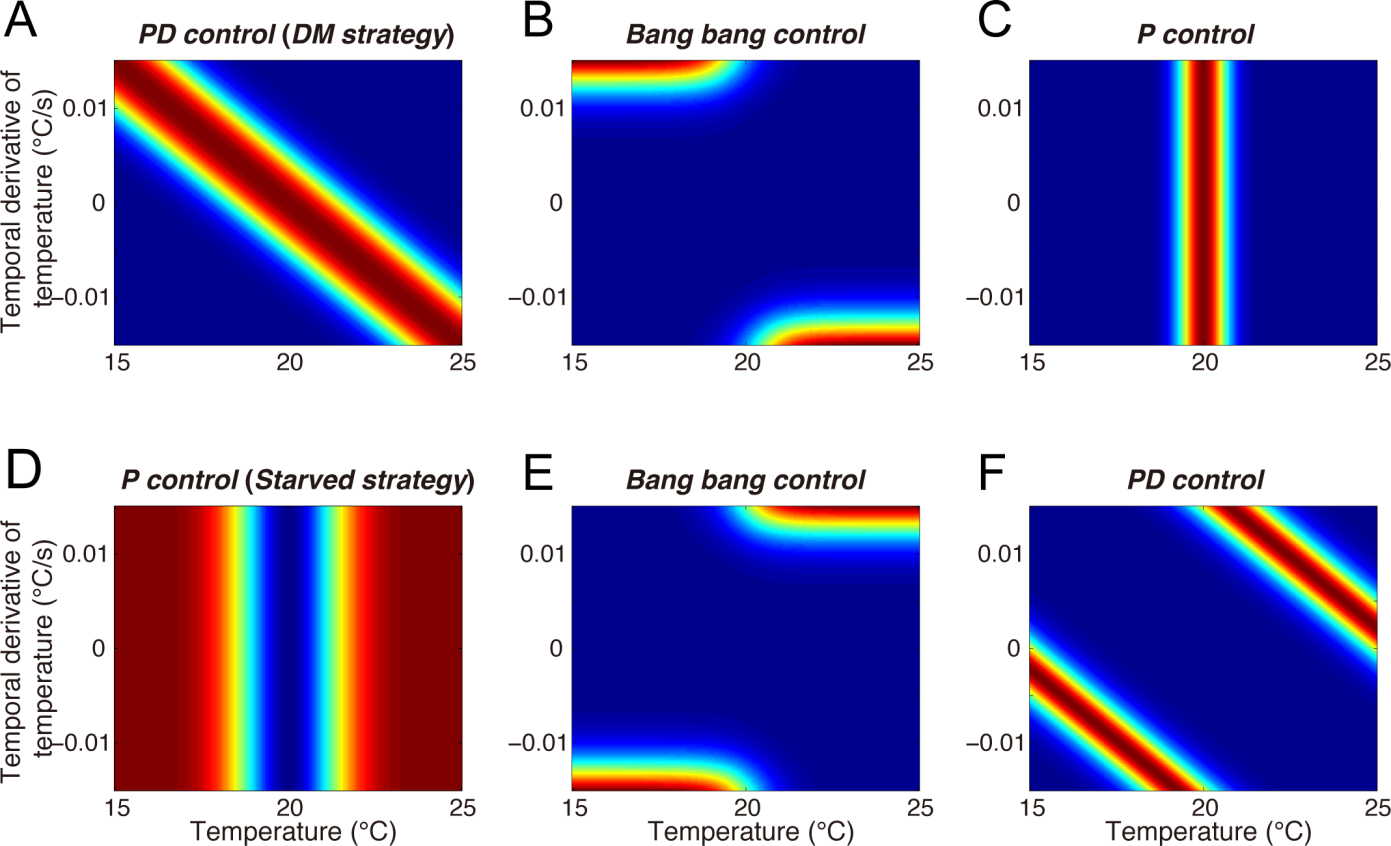
Possible strategies involved in preference and avoidance of the cultivation temperature. Each panel represents the desirability function of a possible strategy (fed worms: **A-C**, starved worms: **D-F**). The prior knowledge that fed worms navigate to the cultivation temperature and starved worms escape the cultivation temperature suggests several possible strategies, but does not identify the actual strategy exhibited by the animals. The inverse reinforcement learning approach identified that the fed worms use the proportional-derivative (PD) control-like DM strategy shown in (A), while the starved worms use the proportional (P) control-like strategy shown in (D).

Further, we performed IRL on behavioral data from starved worms, which were cultivated at 20°C without food and then placed on the thermal gradient. The starved worms dispersed in the low-temperature region and avoided the high-temperature one (**Fig 4Da**). Regarding the desirability function, we found that, compared with the fed worms (**Fig 3B**), the diagonal structure was not present in the starved worms (**Fig 4Db**), suggesting that they did not use DM. In contrast, we could still observe IM (**Fig 5Ab**), indicating that the starved worms retained the ability to perform isothermal tracking. Most importantly, the desirability function was lower at the cultivation temperature than at surrounding temperatures, suggesting that, unlike the fed worms, the starved ones escaped the cultivation temperature region based on sensory information of only the absolute temperature, but not of its temporal derivative. These results indicate that our method could distinguish between strategies of normally fed and starved worms.

## Discussion

In this study, we proposed an IRL framework to identify animal behavioral strategies based on collected behavioral time-series data. We validated the framework using artificial data, and then applied it to behavioral data collected during *C*. *elegans* thermotaxis experiments. We quantitatively identified the thermotactic strategies and discovered that fed worms use both the absolute temperature and its temporal derivative, whereas starved worms only use the absolute temperature. We then visualized the properties of this thermotactic strategy, by means of the desirability function, and successfully identified which states are pleasant and unpleasant for *C*. *elegans*. Finally, we demonstrated the ability of this technique to discriminate alterations in components within a strategy, by using it to compare the desirability functions of two strains of worms with impaired thermosensory neuron function; we found that AFD, but not AWC, neurons are fundamental for the worms to efficiently navigated towards the cultivation temperature.

### Advantages of the IRL approach

Our approach has three advantages. First, it is generally applicable to behavioral data of any organism, not just *C*. *elegans*. Second, it can be applied independently of the experimental conditions. Our approach is especially suitable for analyzing behavior in natural conditions where target animals are behaving freely. To the best of our knowledge, this is the first study to identify the behavioral strategy of a freely-behaving animal by using IRL. Third, this approach estimates the strategy that generates natural behaviors, by introducing passive dynamics in the LMDP. Animal movements are usually restricted by external constraints, including inertia and gravity, as well as by internal (musculoskeletal) constraints; therefore, animals prefer entering a natural unrestricted state-transition. Thus, the LMDP-based IRL is suitable for modeling animal behavioral strategies. Although there are several studies on IRL application to human behaviors [12–15], none of these have considered passive dynamics. Since high-throughput experiments produce massive amounts of behavioral data, our IRL approach could be a fundamental tool for their analysis, with applicability in behavioral sciences, in general, including ecology and ethology.

### Validity of the identified strategies

We applied our IRL approach to worms of different genetic backgrounds (WT and three mutant strains) and confirmed that the identified behavioral strategies undertaken by the animals, as expressed by the desirability function, showed no discrepancy in thermotactic behaviors. The fact that fed WT worms aggregated at the cultivation temperature, while starved WT worms dispersed around it can be explained by the increased and decreased amplitude, respectively, of the desirability function at the cultivation temperature. We found that ASI- and AWC-deficient worms exhibit normal thermotaxis, and their desirability functions were similar to that of WT animals. However, AFD-deficient worms demonstrate cryophilic thermotaxis, consistent with the increased amplitude of the desirability function at lower temperatures. Taken together, these results demonstrate the validity of our approach, as well as its potential to determine changes in behavioral strategies.

### Alternative behavioral strategies

Our approach provides novel insight into how the *C*. *elegans* reaches a target temperature on a thermal gradient. In theory, the strategy we identified is not the sole solution for the animals in order to reach the target state; several alternative solutions could have allowed animals to navigate to their behavioral goals. The strategies undertaken by fed or starved animals and the possible alternative ones are discussed below in terms of control theory [29].

In the case of the fed worms (**Fig 5A–5C**), several alternative strategies might have enabled the animals in their DM towards the goal (cultivation temperature). The DM strategy is shown in **Fig 5A**. **Fig 5B** shows the desirability function for worms switching their preference between a positive and a negative temperature gradient, lower or higher than the goal temperature, representing the so called “bang-bang control”. A previous computational study modeled *C*. *elegans* thermotaxis based on the bang-bang control [30], in which straight runs and random turnings (corresponding to omega and reversal turns) alternate, while the run duration is regulated by the temperature, its temporal derivative and the cultivation temperature. **Fig 5C** shows the resulting desirability function when worms simply prefer the goal temperature, regardless of its temporal derivative. This might be interpreted as “proportional (P) control”. However, the identified DM strategy is based on both the absolute temperature and its temporal derivative, suggesting that the worms in fact perform “proportional-derivative (PD) control”, which is more sophisticated than the bang-bang control.

Regarding the strategy of the starved worms, similar alternatives exist, as discussed above. The worms could escape the cultivation temperature by performing “bang-bang control” or “PD control”, as shown in **Fig 5E and 5F**. The identified starved strategy however is closer to “P control”, which only uses the absolute temperature. Our IRL-based approach is therefore able to clarify how the worms control their thermosensory state throughout migration, which was not understood until now.

### Functional significance of DM and IM strategies

We found that the WT worms use a thermotactic strategy consisting of two components; a diagonal, representing DM; and a horizontal, representing IM. What is the functional meaning of these two strategies? We propose that they might be necessary for balancing exploration and exploitation. Exploitation is the use of pre-acquired knowledge in an effort to obtain rewards, while exploration is the effort of searching for possible greater rewards. For example, worms know that food is associated with the cultivation temperature and can exploit this association. Alternatively, they can explore different temperatures to search for more food than that available at the cultivation temperature. In an uncertain environment, animals usually face an “exploration-exploitation dilemma” [31]; exploitative behaviors reduce the chance to explore for greater rewards, whereas exploratory behaviors disrupt the collection of the already-available reward. Therefore, an appropriate balance between exploration and exploitation is important for controlling behavioral strategies. We propose that DM and IM generate exploitative and explorative behaviors, respectively: the worms, via DM, exploit the cultivation temperature, and at the same time explore possible alternative rewards (food) in different temperatures through IM.

We found that in the case of starved worms, temperature and feeding are dissociated, and worms do not exhibit DM; instead they still exhibit IM. According to these findings, we hypothesize that DM emerges as a consequence of associative learning (association between the cultivation temperature and food access); the IM strategy, however, could be innate. Further investigation regarding these hypotheses should be expected in the future.

In the case of thermosensory neuron-deficient worms, we found that AWC-neuron ablation does not affect the desirability function, whereas AFD-neuron depletion abolishes the DM diagonal component, as well as any bias along the *dT* axis. The AWC and AFD neurons are both known to sense the temporal derivative of temperature, *dT* [16,22,23]. Thus, we can assume that AFD-neuron loss might prevent worms from deciding whether an increase or decrease in temperature is favorable, which could lead to inefficient thermotactic migration. Thus, the AFD, but not AWC neurons, are involved in the DM based on temporal changes in temperature.

### Future perspectives for neuroscience research

Finally, it is worth discussing future perspective of our IRL approach in neuroscience research focusing on higher-order animals beyond *C*. *elegans*. Over the last two decades, several reports have demonstrated that dopaminergic activity in the ventral tegmental area (VTA) encodes for reward prediction error [5], similar to temporal difference (TD) learning in RL [6], suggesting that animal behavioral strategies are associated with reward-based representation. In addition, it is widely believed that RL-like algorithms are processed within functionally connected cortical and subcortical areas, especially within the basal ganglia [32–35] and amygdala [36,37], brain areas that heavily innervated by VTA dopaminergic neurons. Recent advances in neural recording technology have enabled researchers to monitor the activity of neuronal populations related to the reward-based representation of a given strategy in freely-behaving animals. However, the actual rewards for freely-behaving animals, especially those internally-represented in the brain, rather than the primitive ones, like food, are difficult to recognize. Our study shows that the presented IRL framework can identify the reward-based representation of animal strategies, thus allowing the analysis of neural correlates, such as comparing neural activities in freely-behaving animals with strategy-related variables, calculated by using IRL. Therefore, a combination of neuroscience experiments and the IRL technology could contribute in discovering behavioral neural substrates and their computational principles.

## Materials and methods

### Reinforcement learning

RL is a machine learning model that describes how agents learn to obtain an optimal policy, that is, a behavioral strategy, in a given environment [4]. RL consists of several components: an agent, an environment, and a reward function. The agent learns and makes decisions, and the environment is defined by everything else. The agent continuously interacts with the environment, in which the state of the agent changes based on its actions (behavior), and the agent gets a reward at the new state according to the reward function. The aim of the agent is to identify an optimal strategy (policy) that maximizes cumulative rewards in the long term.

In this study, the environment and the agent’s behavioral strategy were modeled as an LMDP, one of settings of RL [20]. The LMDP included the passive dynamics of the environment, in the absence of control, and the controlled dynamics that reflect a behavioral strategy. Passive and controlled dynamics were each defined by transition probabilities from state *s* to *s*’, namely, *p*(*s*’|*s*) and *π*(*s*’|*s*), respectively. In each state, the agent not only acquires a reward, but also receives resistance to passive dynamics (**Fig 1C**). Thus, the net reward is described as

l(s,π(⋅|s))=r(s)−KL[π(⋅|s)‖p(⋅|s)], (2)

where *r*(*s*) denotes a state reward and *KL*[*π*(.|*s*)||*p*(.|*s*)] indicates the Kullback–Leibler (KL) divergence between *π*(.|*s*) and *p*(.|*s*), which represents the resistance to passive dynamics. The optimal policy that maximizes the cumulative net reward has been analytically obtained [20] as

$$\pi^{*}(s^{\prime }|s)=\frac{p(s^{\prime }|s)\exp(v(s^{\prime }))}{\sum_{y}p(y|s)\exp(v(y))},$$ (3)

where the asterisk indicates optimal, and *v*(*s*) is the value function, i.e., the cumulative net reward expected from state *s* toward the future:

$$v(s)=E\left[\sum_{t}l\left(s,\pi^{*}(\cdot |s)\right)|s_{t}=s\right].$$ (4)

Here, we briefly show how to derive Eq (3). First, the controlled dynamics were defined as

$$\pi (s^{\prime }|s;\;u)=p(s^{\prime }|s)\exp(u_{s^{\prime }}),$$ (5)

where the elements *u*_*s*_ of a vector **u** directly modulate the transition probability of passive dynamics. Note that *π*(*s*’|*s*, **0**) = *p*(*s’*|*s*). Because of probability, Eq (5) must satisfy

$$\sum_{s^{\prime }}\pi (s^{\prime }|s;\;u)=1.$$ (6)

The value function can be rewritten by the Bellman equation:

$$v(s)=\underset{u}{\max}\left\{l(s,u)+\sum_{s^{\prime }}\pi (s^{\prime }|s;\;u)v(s^{\prime })\right\},$$ (7)

where l(s,u)=l(s,π(⋅|s;u)). The maximization in Eq (7), subjected to Eq (6) by the method of Lagrange multipliers, yields u^*^, which represents the optimal strategy. Substituting u^*^ in Eq (5) gives Eq (3). In addition, substituting the optimal strategy [Eq (3)] in the Bellman Eq (7) and dropping the max operator lead to

$$\exp(v(s))=\exp(r(s))\sum_{s^{\prime }}p(s^{\prime }|s)\exp(v(s^{\prime })),$$ (8)

which satisfies Bellman’s self-consistency. Using this equation, *v*(*s*) can be calculated from the reward function *r*(*s*), and vice versa. The full derivation is described in [20].

### Inverse reinforcement learning (estimation of the value function)

To estimate *v*(*s*), we assumed that the observed sequential state transitions {*s*_*t*_, *s*_*t*+1_}_*t* = 1:*T*_ are generated by the stationary optimal policy π^*^. We then maximized the likelihood of the sequential state transition:

$$L[v(s)]=\prod_{t}\pi^{*}(s_{t+1}|s_{t};\;v(s)),$$ (9)

where π^*^(*s*_*t*+1_|*s*_*t*_; *v*(*s*)) corresponds to Eq (3). This estimation is called OptV [21]. Based on the estimated *v*(*s*), the primary reward function, *r*(*s*), can be calculated by using Eq (8).

In our implementation, states were represented by a tabular format, in which 2-D space (temperature and its temporal derivative) was divided as a mesh grid. Thus, our IRL required a number of state trajectory data, spanning the entire mesh grid. In order to compensate for data limitation and noisy sensory systems, we assumed that animals have value functions that are smooth in their state space. To obtain smooth value functions, we regularized MLE as

$$\hat{v}(s)=\underset{v(s)}{\arg\;\max}\left[\log L(v(s))-\lambda \sum_{s}\sum_{s^{\prime }\in \chi (s)}{\left|v(s)-v(s^{\prime })\right|}^{2}\right],$$ (10)

where the first term represents the log-likelihood and the second term represents a smoothness constraint introduced to the value function; a positive constant *λ* indicates the strength of the constraint, and *χ*(*s*) indicates a set of neighboring states of *s* in the state space. The evaluation function, i.e., the regularized log-likelihood, is convex with respect to *v*(*s*), which means there are no local minima in its optimization procedure.

### Passive dynamics of thermotaxis in *C. elegans*

To apply the LMDP-based IRL to the thermotactic behaviors of *C*. *elegans*, state *s* and passive dynamics *p*(*s*’|*s*) were defined (phase 2 and 3 in **Fig 1D**). We previously found that the thermosensory AFD neurons encode the temporal derivative of the environmental temperature [22] and thus assumed that worms can sense not only the absolute temperature, *T*, but also its temporal derivative, *dT/dt*. We therefore set a 2-D state representation as (*T*, *dT*). For simplicity *dT/dt* is simply denoted as *dT*.

The passive dynamics were described by the transition probability of a state (*T*, *dT*) as

$$P\left((T^{\prime },dT^{\prime })|\left(T,dT\right)\right)=N\left(T^{\prime }|T+dT\Delta t,\sigma_{T}\right)N\left(dT^{\prime }|dT,\sigma_{dT}\right),$$ (11)

where *N*(*x*|*μ*, σ) indicates a Gaussian distribution of variable *x* with mean *μ* and variance σ, and *Δt* indicates the time interval of monitoring during behavioral experiments. The passive-dynamics aspect can be loosely interpreted as if the worms inertially migrate in a short time interval under a thermal gradient, and may be perturbed by white noise. The distribution of passive dynamics can be arbitrary selected, and the choice of Gaussian was not due to mathematical necessity for the IRL.

### Artificial data

To confirm that our regularized version of OptV (Eq (6)) provided a good estimation of the value function, we used simulation data. First, we designed the value function of *T* and *dT* as the ground truth (**S1A Fig**), and passive dynamics through Eq (7). Thus, the optimal policy was defined by Eq (3). Second, we generated a time-series of state transitions based on the optimal policy and separated these time series into training and test datasets. Next, we estimated *v*(*s*) from the training dataset, varying the regularization parameter *λ* in Eq (6) (**S1B Fig**). We then evaluated the squared error between the behavioral strategy, based on the ground truth, and the estimated *v*(*s*), using the test dataset. Since the squared error on the test data was substantially reduced (by 88.1%) due to regularization, we deemed it effective for avoiding overfitting (**S1C Fig**).

### Cross-validation

For estimating *v*(*s*), we performed cross-validation to determine *λ* in Eq (10), and *σ*_*T*_ and *σ*_*dT*_ in Eq (11), with which the prediction ability is maximized. We divided the time-series behavioral data equally into nine parts. We then independently performed estimation of *v*(*s*) nine times; for each estimation, eight of the nine parts of the data were used for estimation, while the remaining part was used to evaluate the prediction ability of the estimated value function by the likelihood [Eq (9)]. We then optimized those parameters at which we obtained the highest log-likelihood, as averaged from the nine estimations.

### Surrogate method-based statistical testing

To check whether the DM and IM strategies were not obtained by chance, surrogate method-based statistical testing was performed under a null hypothesis that the worms randomly migrated under a thermal gradient with no behavioral strategy. We first constructed a set of artificial temperature time-series, which could be observed under the null hypothesis. By using the iterated amplitude adjusted Fourier transform method [38], we prepared 1000 surrogate datasets by shuffling the observed temperature time-series (**S5A Fig**), while preserving the autocorrelation of the original time-series (**S5B Fig**). We then applied our IRL algorithm to this surrogate dataset to estimate the desirability function (**S5C Fig**). To assess the significance of the DM and IM strategies, we calculated the sums of the estimated desirability functions within the previously described horizontal and diagonal regions, respectively (**S5D Fig**). Empirical distributions of these test statistics for the surrogate datasets could then serve as null distributions (**S5E Fig**). For both DM and IM, the test statistic derived using the original desirability function was located above the empirical null distribution (*p* <0.001 for the DM strategy; *p* <0.001 for the IM strategy), indicating that both strategies were not obtained by chance but reflected an actual strategy of thermotaxis.

### *C. elegans* preparation

All worms were hermaphrodites and cultivated on OP50 as bacterial food using standard techniques [39]. The following strains were used: N2 wild-type Bristol strain, PY7505 *oyIs84[gcy-27p*::*cz*::*caspase-3(p17)*, *gpa-4p*::*caspase-3(p12)*::*nz*, *gcy-27p*::*GFP*, *unc-122p*::*dsRed]*, IK2808 *njIs79[ceh-36p*::*cz*::*caspase-3(p17)*, *ceh-36p*::*caspase-3(p12)*::*nz*, *ges-1p*::*NLS*::*GFP]* and IK2809 *njIs80[gcy-8p*::*cz*::*caspase-3(p17)*, *gcy-8p*::*caspase3(p12)*::*nz*, *ges-1p*::*NLS*::*GFP]*. The ASI-ablated strain (PY7505) was a kind gift from Dr. Piali Sengupta [27]. The AFD-ablated strain (IK2809) and the AWC-ablated strain (IK2808) were generated by the expression of reconstituted caspases [40]. Plasmids carrying the reconstituted caspases were injected at 25 ng/μl with the injection marker pKDK66 (*ges-1p*::*NLS*::*GFP*) (50 ng/μl). Extrachromosomal arrays were integrated into the genome by gamma irradiation, and the resulting strains were outcrossed four times before analysis. To assess the efficiency of cell killing by the caspase transgenes, the integrated transgenes were crossed into integrated reporters that expressed GFPs in several neurons, including the neuron of interest, as follows: IK0673 *njIs2*[*nhr-38p*::*GFP*, *AIYp*::*GFP*] for AFD and IK2811 *njIs82*[*ceh-36p*::*GFP*, *glr-3p*::*GFP*] for AWC. Neuronal loss was confirmed by the disappearance of fluorescence; 100% of *njIs80* animals displayed a loss of AFD and 98.4% of the *njIs79* animals displayed a loss of AWC neurons.

### Thermotaxis assay

Thermotaxis assays were performed as previously described [41]. Animals were first cultivated at 20°C and then placed on the center of an assay plate (14 cm × 10 cm, 1.45 cm height) containing 18 ml of thermotaxis medium, supplemented with 2% agar, and were allowed to move freely for 60 min. The center of the plate was adjusted to 17, 20, or 23°C, to create three different gradient conditions, and the plates were then maintained at a linear thermal gradient of approximately 0.45°C/cm.

### Behavioral recording

Worm behaviors were recorded using a CMOS sensor camera-link camera (8 bits, 4,096 × 3,072 pixels; CSC12M25BMP19-01B; Toshiba-Teli), a Line-Scan Lens (35 mm, f/2.8; YF3528; PENTAX), and a camera-link frame grabber (PCIe-1433; National Instruments). The camera was mounted at a distance above the assay plate and consistently produced an image with 33.2 μm per pixel. The frame rate of recordings was approximately 13.5 Hz. Images were captured and processed by a multi-worm Tracker [24], to detect worm bodies and measure the position of the centroid.

## Supporting information

S1 Fig Validation of the regularized (OptV) estimation method by using artificial data. **(A)** The desirability function corresponding to the ground truth value function used for generation of artificial data. Time-series data were artificially generated as training and test data sets by sampling Eq (1), based on the ground truth of the value function. **(B)** The desirability functions under three different regularization parameters (*λ*) were visualized from the estimated value functions. **(C)** Squared error between the behavioral strategies based on the ground truth and estimated value functions using the test data set. The presence of an optimal *λ*, at which the minimal squared error is obtained, indicates that the regularization was effective for accurately estimating the value function.
(TIF)

S2 Fig Behaviors in the *T*-*dT* space. **(A)** *T-dT* trajectories of fed WT worms. This is another representation of Fig 2E. **(B)** Distributions of *T* and *dT* in all trajectories of fed WT worms. Notice that the distribution is substantially different from the desirability function (see Fig 3B). **(C)** Scatter plot of *T* and *dT* at 5 seconds before the moment of sharp turns. Correlation coefficient was 3.6e-10. Note that *dT* is 0 at the moment of a sharp turn, because the worm stops in order to make large directional changes. **(D)** Histogram of the scatter plot in C.
(TIF)

S3 Fig Inverse reinforcement learning analysis using one-dimensional state representation. IRL was performed with one-dimensional state representation (*s* = (*T*)). **(A)** The desirability function was calculated using the estimated value function. In the estimation, the regularization parameter, *λ*, in Eq (6), was optimized by cross-validation. **(B)** The prediction ability was compared between IRLs with *s* = (*T*, *dT*) and *s* = (*T*) using a cross-validation dataset. The negative log-likelihood of behavioral strategies (Eq (1)) when estimating the value function of both *T* and *dT* (see Fig 3B), was significantly smaller than when estimating the value function of *T* alone (A; *p =* 0.0002; Mann-Whitney U test). Thus, the behavioral strategy with *s* = (*T*, *dT*) was more appropriate than that with *s* = (*T*). **(C)** The desirability function became smoother as *λ* increased, with a peak around the cultivation temperature (20°C).
(TIF)

S4 Fig Reproduction of thermotaxis by simulating the identified strategy. **(A)** The identified desirability function of the fed WT worms. This is identical to Fig 3B. **(B)** Temperature time-series of simulated worms started from 15, 20, or 25°C with 0°C/s. In the simulation, the state transition was sampled from Eq (3) using the identified desirability function in (A). Different colored lines correspond to different simulation runs. **(C)** Temporal changes in distributions of 100 simulated worms. Notice that most worms converged around the cultivation temperature, i.e., 20°C.
(TIF)

S5 Fig Statistical test of the behavioral strategy reliability using the surrogate method. The reliability of the directed migration (DM) and isothermal migration (IM) strategies (see Fig 3) was assessed by means of statistical testing with the null hypothesis that worms randomly migrate with no behavioral strategy. **(A)** To generate time-series data under this null hypothesis, original time-series data of temperature (left panel) were surrogated by the iterated amplitude adjusted Fourier transform method (right panel). **(B)** Before and after the surrogation, the autocorrelations were almost preserved. **(C)** The desirability functions estimated from the surrogate datasets. **(D)** The DM and IM strategies correspond to the red-highlighted diagonal and horizontal regions of the desirability function, respectively. Within these regions, sums of the estimated desirability functions were calculated as test statistics. **(E)** Histograms of the empirical null distributions of the test statistics for the DM and IM strategies. Test statistics derived by the original desirability function (red arrows) are located above the empirical null distributions (*p* <0.001 for the PT strategy; *p* <0.001 for the IT strategy).
(TIF)

S6 Fig Estimated value/reward functions and state distributions. The estimated value functions, reward functions, and state distributions are depicted for the ASI- **(A)**, AWC- **(B)**, and AFD-deficient worms **(C)**, as well as for the starved WT worms **(D)**.
(TIF)
