# Supplementary figures and images for: Identification of animal behavioral strategies by inverse reinforcement learning
Source: PLoS Comput Biol. 2018 May 2;14(5):e1006122. doi: 10.1371/journal.pcbi.1006122 (PMC5951592; doi:10.1371/journal.pcbi.1006122)

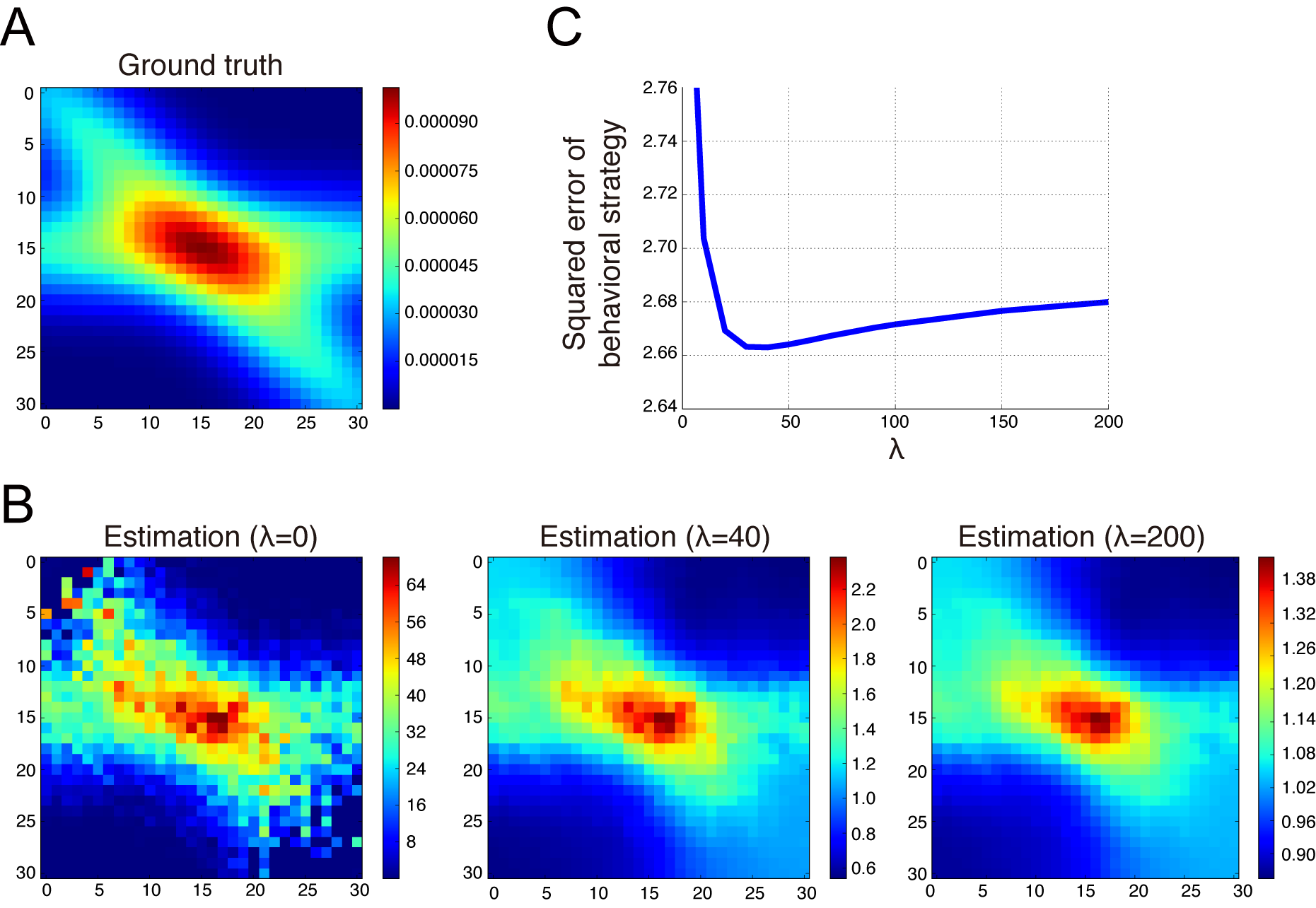

Supplement: S1 Fig — (A) The desirability function corresponding to the ground truth value function used for generation of artificial data. Time-series data were artificially generated as training and test data sets by sampling Eq (1), based on the ground truth of the value function. (B) The desirability functions under three different regularization parameters (λ) were visualized from the estimated value functions. (C) Squared error between the behavioral strategies based on the ground truth and estimated value functions using the test data set. The presence of an optimal λ, at which the minimal squared error is obtained, indicates that the regularization was effective for accurately estimating the value function. (TIF) [file pcbi.1006122.s001.tif]

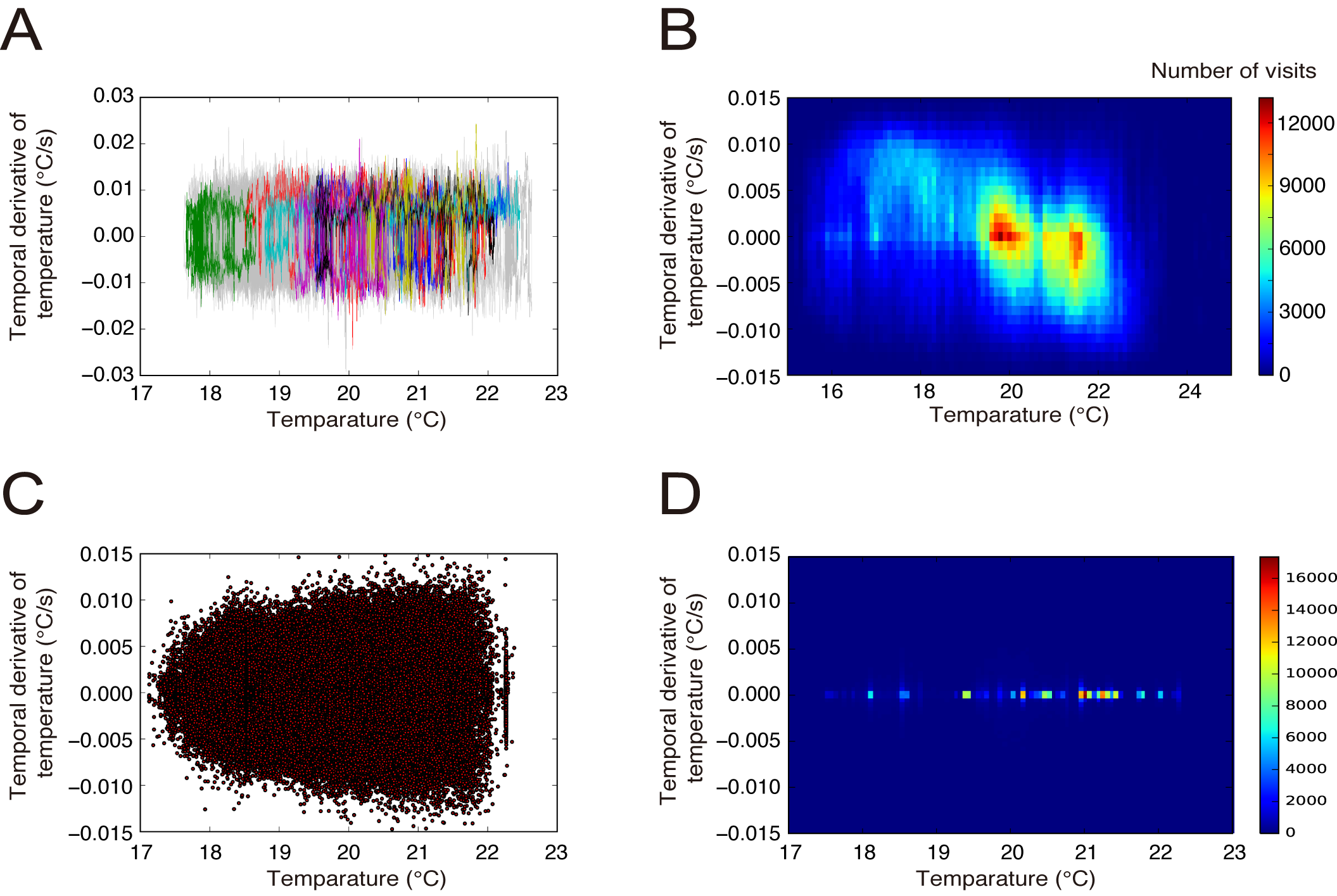

Supplement: S2 Fig — (A) T-dT trajectories of fed WT worms. This is another representation of Fig 2E. (B) Distributions of T and dT in all trajectories of fed WT worms. Notice that the distribution is substantially different from the desirability function (see Fig 3B). (C) Scatter plot of T and dT at 5 seconds before the moment of sharp turns. Correlation coefficient was 3.6e-10. Note that dT is 0 at the moment of a sharp turn, because the worm stops in order to make large directional changes. (D) Histogram of the scatter plot in C. (TIF) [file pcbi.1006122.s002.tif]

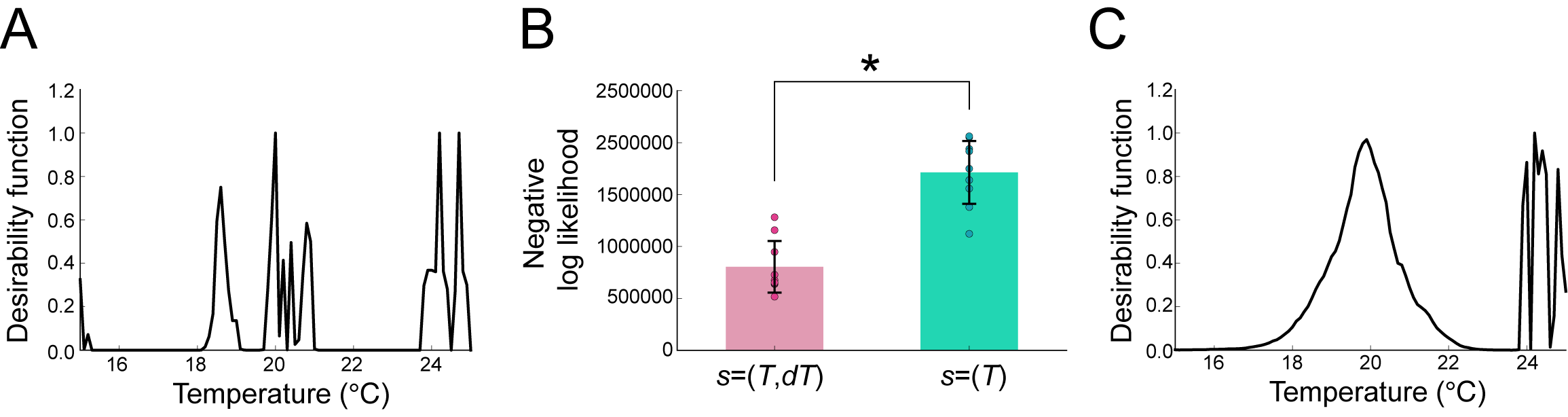

Supplement: S3 Fig — IRL was performed with one-dimensional state representation (s = (T)). (A) The desirability function was calculated using the estimated value function. In the estimation, the regularization parameter, λ, in Eq (6), was optimized by cross-validation. (B) The prediction ability was compared between IRLs with s = (T, dT) and s = (T) using a cross-validation dataset. The negative log-likelihood of behavioral strategies (Eq (1)) when estimating the value function of both T and dT (see Fig 3B), was significantly smaller than when estimating the value function of T alone (A; p = 0.0002; Mann-Whitney U test). Thus, the behavioral strategy with s = (T, dT) was more appropriate than that with s = (T). (C) The desirability function became smoother as λ increased, with a peak around the cultivation temperature (20°C). (TIF) [file pcbi.1006122.s003.tif]

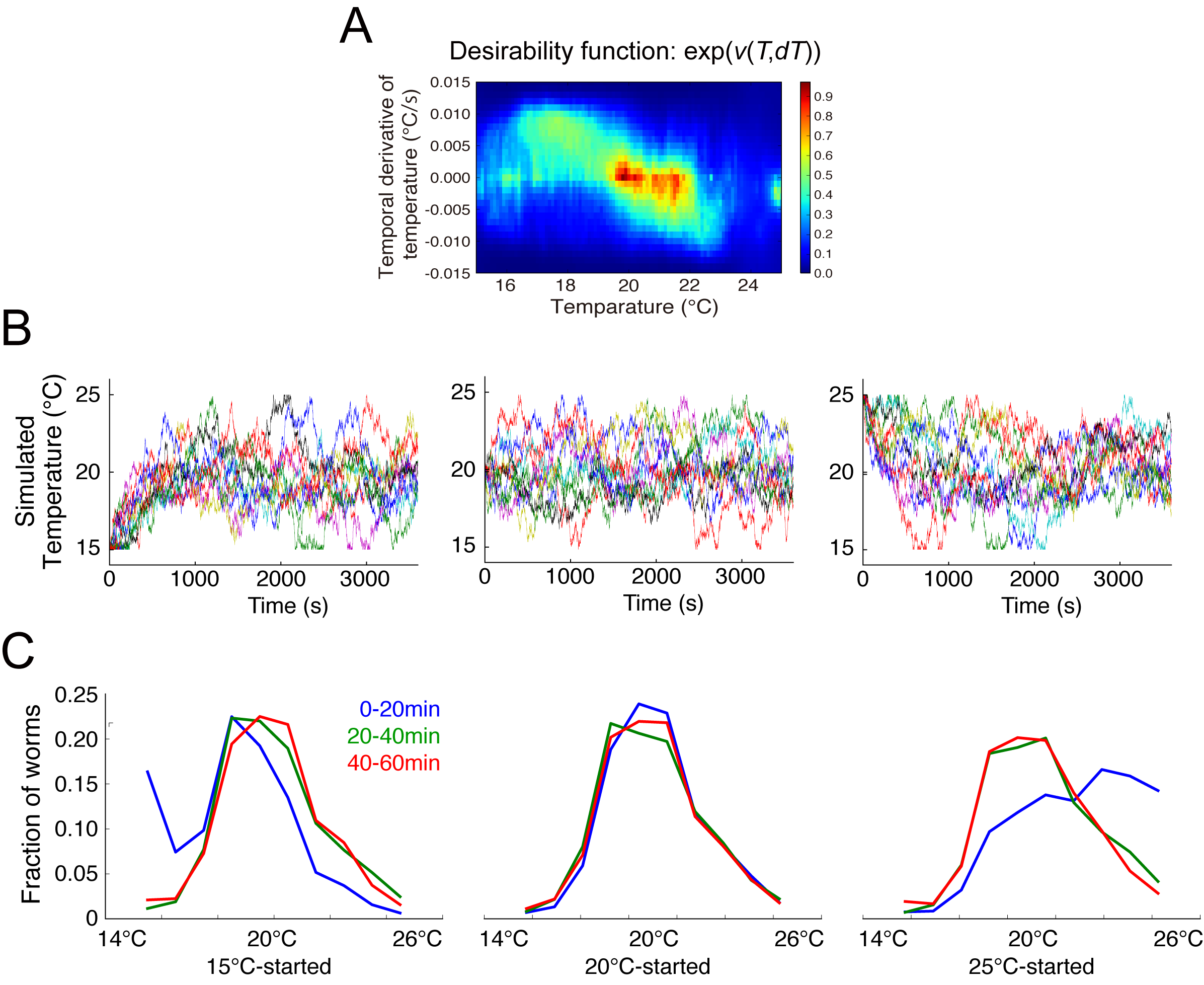

Supplement: S4 Fig — (A) The identified desirability function of the fed WT worms. This is identical to Fig 3B. (B) Temperature time-series of simulated worms started from 15, 20, or 25°C with 0°C/s. In the simulation, the state transition was sampled from Eq (3) using the identified desirability function in (A). Different colored lines correspond to different simulation runs. (C) Temporal changes in distributions of 100 simulated worms. Notice that most worms converged around the cultivation temperature, i.e., 20°C. (TIF) [file pcbi.1006122.s004.tif]

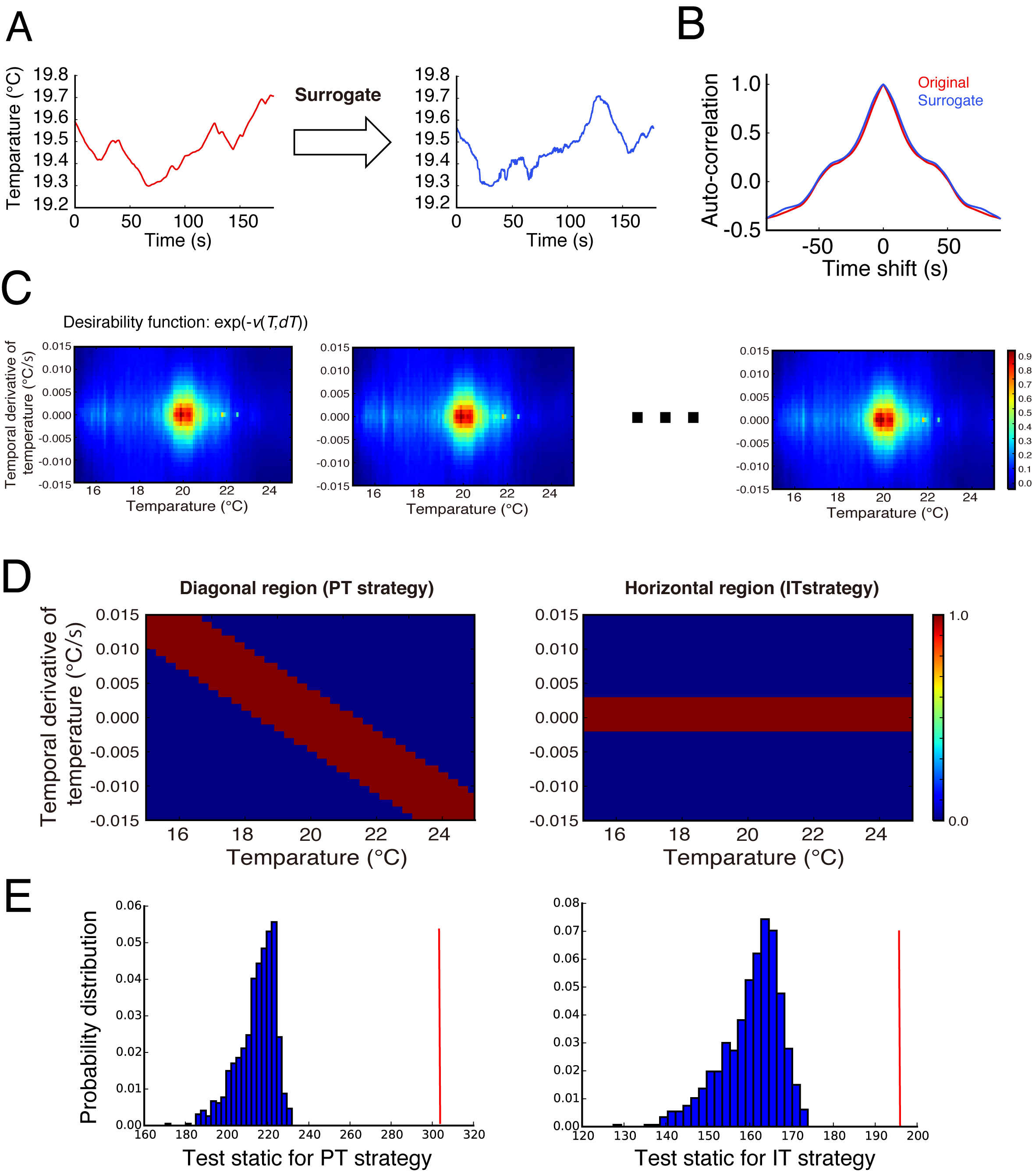

Supplement: S5 Fig — The reliability of the directed migration (DM) and isothermal migration (IM) strategies (see Fig 3) was assessed by means of statistical testing with the null hypothesis that worms randomly migrate with no behavioral strategy. (A) To generate time-series data under this null hypothesis, original time-series data of temperature (left panel) were surrogated by the iterated amplitude adjusted Fourier transform method (right panel). (B) Before and after the surrogation, the autocorrelations were almost preserved. (C) The desirability functions estimated from the surrogate datasets. (D) The DM and IM strategies correspond to the red-highlighted diagonal and horizontal regions of the desirability function, respectively. Within these regions, sums of the estimated desirability functions were calculated as test statistics. (E) Histograms of the empirical null distributions of the test statistics for the DM and IM strategies. Test statistics derived by the original desirability function (red arrows) are located above the empirical null distributions (p <0.001 for the PT strategy; p <0.001 for the IT strategy). (TIF) [file pcbi.1006122.s005.tif]

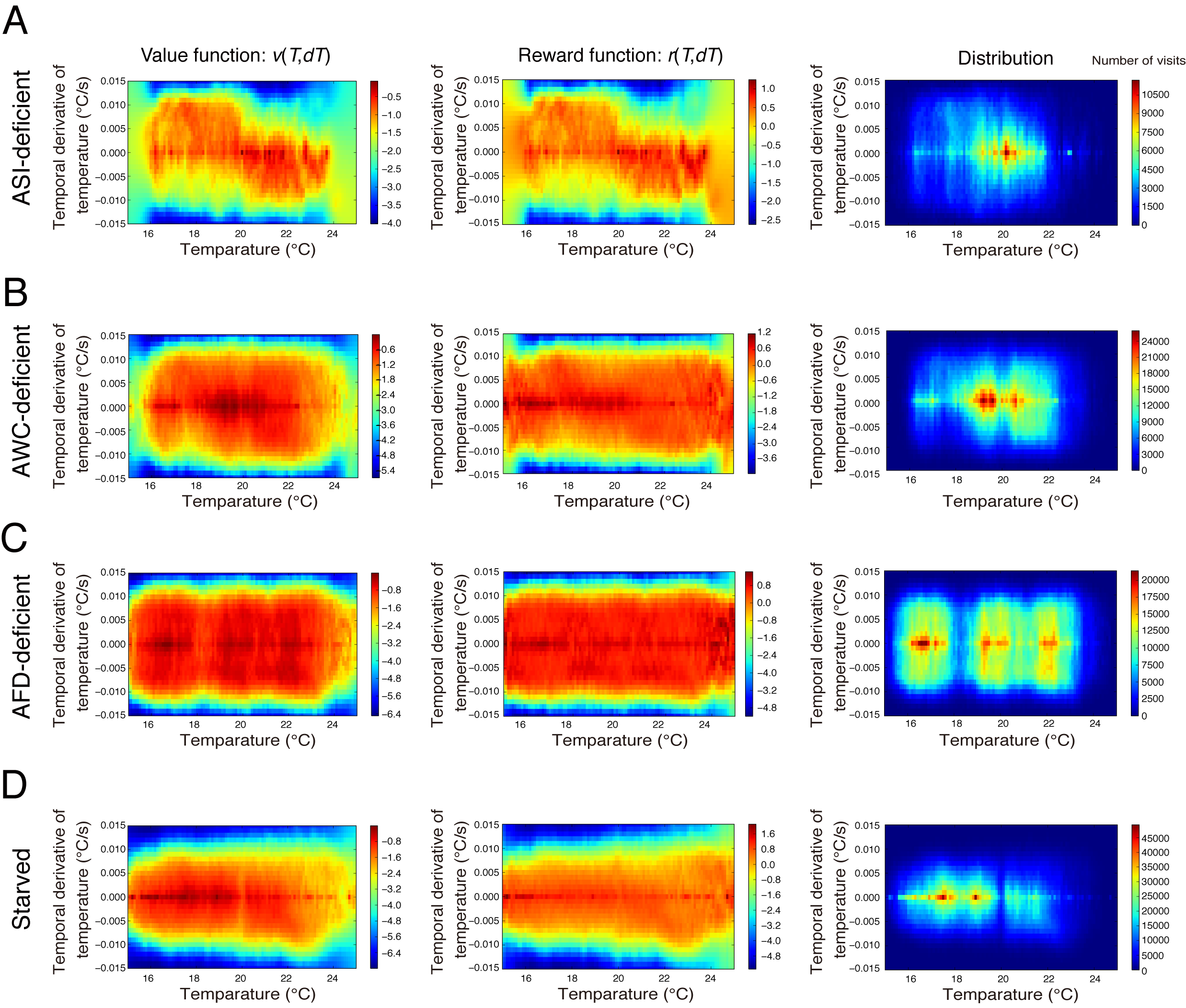

Supplement: S6 Fig — The estimated value functions, reward functions, and state distributions are depicted for the ASI- (A), AWC- (B), and AFD-deficient worms (C), as well as for the starved WT worms (D). (TIF) [file pcbi.1006122.s006.tif]
